# Metabolic adaptations driving innate immune memory: mechanisms and therapeutic implications

**DOI:** 10.1093/jleuko/qiaf037

**Published:** 2025-03-26

**Authors:** Dan Hao, Margaret A McBride, Julia K Bohannon, Antonio Hernandez, Benjamin Klein, David L Williams, Edward R Sherwood

**Affiliations:** Department of Anesthesiology, Vanderbilt University Medical Center, 1211 Medical Center Drive, Nashville, TN 37232, United States; Department of Pathology, Microbiology and Immunology, Vanderbilt University Medical Center, 1211 Medical Center Drive, Nashville, TN 37232, United States; Department of Anesthesiology, Vanderbilt University Medical Center, 1211 Medical Center Drive, Nashville, TN 37232, United States; Department of Pathology, Microbiology and Immunology, Vanderbilt University Medical Center, 1211 Medical Center Drive, Nashville, TN 37232, United States; Department of Anesthesiology, Vanderbilt University Medical Center, 1211 Medical Center Drive, Nashville, TN 37232, United States; Department of Anesthesiology, Vanderbilt University Medical Center, 1211 Medical Center Drive, Nashville, TN 37232, United States; Department of Surgery, East Tennessee State University, Quillen College of Medicine, P.O. Box 70575, Johnson City, TN 37614, United States; Center for Inflammation, Infectious Disease and Immunology, Quillen College of Medicine, 1276 Gilbreath Drive, Johnson City, TN 37614, United States; Department of Anesthesiology, Vanderbilt University Medical Center, 1211 Medical Center Drive, Nashville, TN 37232, United States; Department of Pathology, Microbiology and Immunology, Vanderbilt University Medical Center, 1211 Medical Center Drive, Nashville, TN 37232, United States; Department of Surgery, East Tennessee State University, Quillen College of Medicine, P.O. Box 70575, Johnson City, TN 37614, United States; Center for Inflammation, Infectious Disease and Immunology, Quillen College of Medicine, 1276 Gilbreath Drive, Johnson City, TN 37614, United States

**Keywords:** immune therapy, innate immune memory, metabolism, trained immunity

## Abstract

Immune memory is a hallmark of the adaptive immune system. However, recent research reveals that innate immune cells also retain memory of prior pathogen exposure that prompts enhanced responses to subsequent infections. This phenomenon is termed “innate immune memory” or “trained immunity.” Notably, remodeling of cellular metabolism, which closely links to epigenetic reprograming, is a prominent feature of innate immune memory. Adaptations in glycolysis, the tricarboxylic acid cycle, oxidative phosphorylation, glutaminolysis, and lipid synthesis pathways are critical for establishing innate immune memory. This review provides an overview of the current understanding of how metabolic adaptations drive innate immune memory. This understanding is fundamental to understanding innate immune system functions and advancing therapies against infectious diseases.

## Teaser

Myeloid cells undergo metabolic adaptations in response to encounters with microbes or microbe-derived ligands to drive an augmented antimicrobial phenotype.

## Key Concepts

- Innate immune memory is defined as the ability of innate leukocytes to develop memory of prior pathogen exposure
- Epigenetic and metabolic adaptations drive innate immune memory
- Memory innate leukocytes mediate an augmented response to subsequent infection
- Innate immune memory may underlie some chronic inflammatory conditions such as atherosclerosis

## Open Questions

- How do metabolic adaptations drive the augmented killing phenotype of memory myeloid cells?
- Do different myeloid cell populations use common or differing metabolic adaptations to drive the memory phenotype?
- Can metabolic adaptations be targeted for therapeutic benefit?

## 1. Introduction

The immune system consists of innate and adaptive branches. In the context of antimicrobial responses, the innate immune system provides a rapid, nonspecific response to pathogens. The adaptive system, though slower, has high specificity and the ability to develop long-lasting immunological memory of prior pathogen exposure.^1,2^ However, recent evidence reveals that innate immune cells also retain memory of prior encounters with microbes and pathogen-associated molecular patterns (PAMPs), leading to enhanced broad-spectrum antimicrobial responses—a phenomenon termed “innate immune memory” or “trained immunity”.^3^ Various immunological adaptations facilitate innate immune memory, which has the potential to enable or combat a multitude of diseases by modulating innate leukocyte functions.^4–10^

**Fig. 1.**
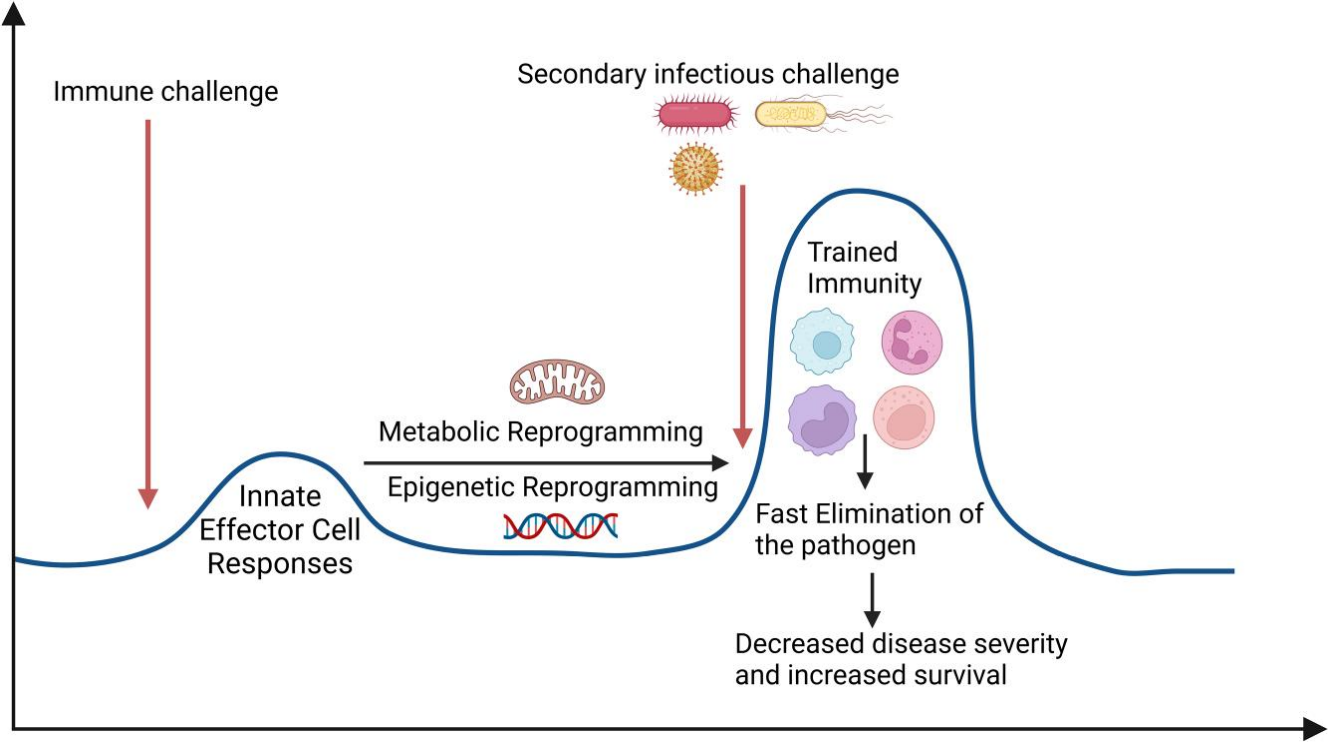
The biogenesis of trained immunity. Activation of the innate immune system triggers inflammation, metabolic adaptations, and epigenetic reprograming with subsequent augmentation of the antimicrobial response to secondary infections.

Induction of innate immune memory involves epigenetic and metabolic reprograming of innate leukocytes to enhance antimicrobial functions^11,12^ (Fig. 1). These changes include stable chromatin modifications in gene regulatory regions, which prime transcriptional responses to subsequent stimuli.^13^ Trained immune cells also undergo metabolic pathway rewiring, which is vital for enhanced functionality. These pathways not only provide energy and molecular building blocks but also function as signaling agents that regulate the immune response.^14,15^ Moreover, various metabolic intermediates can influence epigenetic enzyme activity by acting as substrates or cofactors, which provides an important connection between metabolic and epigenetic processes.^16^ Recent findings have underscored the importance of specific metabolic adaptations in shaping the innate memory phenotype. In this review, we will summarize these findings and offer insights into the metabolic adaptations that drive innate immune memory, along with their therapeutic implications.

### 1.1 Basis of trained immunity

Innate immune memory refers to the ability of the innate immune system to develop memory of prior pathogen exposure and to enhance responsiveness to subsequent infections^17^ (Fig. 1). It is particularly significant for organisms lacking an adaptive immune system, such as invertebrates and plants.^3–5,18,19^ Infection, exposure to microbe-derived PAMPs or treatment with synthetic pattern recognition receptor ligands triggers the innate immune memory response. Importantly, this increased responsiveness to infection is not specific to the initial stimulus. For example, treatment with toll-like receptor 4 (TLR4) ligands, such as lipopolysaccharide (LPS) or monophosphoryl lipid A (MPLA), derived from Gram-negative bacteria, imparts resistance to a broad range of pathogens including Gram-negative (*Pseudomonas aeruginosa*), Gram-positive (*Staphylococcus aureus*), and fungal (*Candida albicans*) species, as well as polymicrobial infection caused by cecal ligation and puncture.^10,20–22^ Innate myeloid cells drive the augmented antimicrobial response, which operates independently of the adaptive immune system.^9,10^ Ligands that activate toll-like receptor 2 (TLR2) (peptidoglycan), toll-like receptor 9 (TLR9) (cytosine-phosphorothioate-guanine oligodeoxynucleotides; CpG-ODN) or dectin-1 (β-glucan) are also highly effective at inducing innate immune memory and broad-spectrum resistance to infection.^9,11,23^

The broad, nonspecific nature of innate immune memory has potential clinical implications. MPLA and CpG-ODN are highly effective vaccine adjuvants and other immune training reagents are being investigated for this application.^24,25^ The wide-ranging defense provided by innate immune training also holds promise in managing infections among critically ill, hospitalized and immunosuppressed patients. For example, vaccination with Bacillus Calmette–Guérin (BCG) not only provides targeted protection against *Mycobacterium tuberculosis* infection but also protects against other respiratory pathogens and neonatal sepsis.^26,27^ Hospital-acquired infections are especially burdensome, impacting approximately 2 million patients and causing at least 90,000 premature deaths every year in the United States.^28,29^ Antibiotic resistance further complicates treatment.^30,31^ Severe infections commonly progress to sepsis, a systemic response to infection that leads to organ dysfunction and is the leading cause of death in noncardiac intensive care units.^32,33^ Sepsis has proven exceedingly difficult to treat and patients that survive sepsis suffer long-term physical and cognitive disabilities and a high 1-year mortality rate.^34–36^ Immunotherapy that augments innate host resistance to infections provides a way of decreasing the incidence and severity of severe infections and sepsis.

**Fig. 2.**
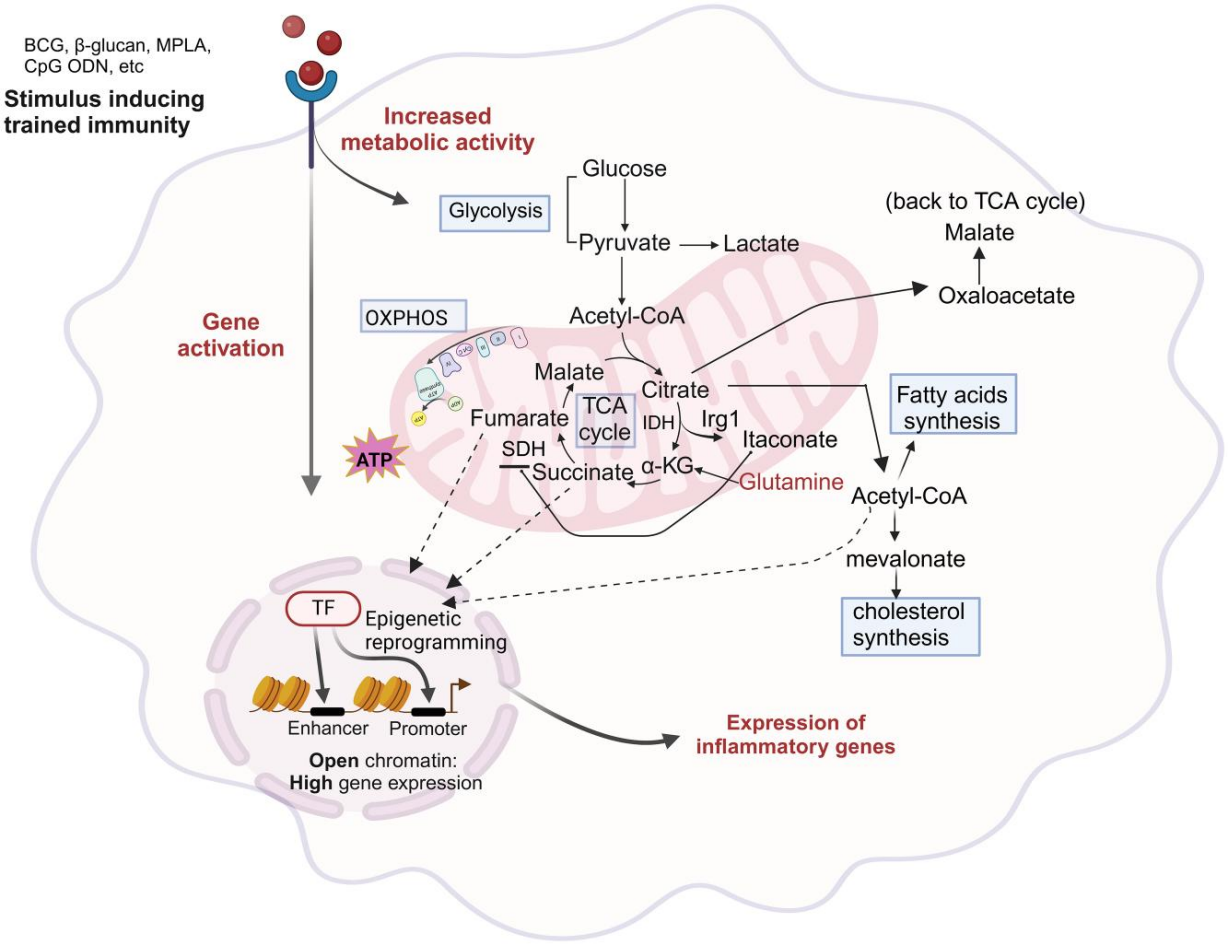
Overview of metabolic remodeling in trained immunity. The induction, regulation, and maintenance of trained immunity rely on coordinated activity of various metabolic pathways, including glycolysis, OXPHOS, glutaminolysis, cholesterol, and fatty acid synthesis. These pathways produce crucial metabolites that drive epigenetic and functional adaptations. A hallmark of trained immunity metabolism is enhanced glycolysis, resulting in lactate production. Glutamine metabolism fuels the TCA cycle, resulting in the accumulation of key metabolites. A break in the TCA cycle distal to citrate, with restored flux at α-ketoglutarate, promotes itaconateproduction, cholesterol and fatty acid synthesis. Accumulated metabolites like succinate, fumarate, and acetyl-CoA contribute to epigenetic changes by modulating histone and DNA methylation. BCG, Bacillus Calmette–Guerin; MPLA, monophosphoryl lipid A; CpG-ODN, cytosine-phosphorothioate-guanine oligodeoxynucleotides; TCA, tricarboxylic acid (Cycle); OXPHOS, oxidative phosphorylation; IDH, isocitrate dehydrogenase; Irg1, immunoresponsive gene 1; SDH, succinate dehydrogenase; α-KG, alpha-ketoglutarate.

Mounting evidence connects trained immunity to metabolic remodeling in innate leukocytes. Central metabolic pathways such as glycolysis, the tricarboxylic acid cycle (TCA cycle), oxidative phosphorylation (OXPHOS), glutaminolysis, fatty acid oxidation (FAO), and cholesterol biosynthesis play key roles in this process (Fig. 2). Understanding and targeting these metabolic alterations presents exciting opportunities for developing innovative therapeutic strategies for vulnerable populations.

### 1.2 Reprograming metabolic pathways to drive innate immune memory

Metabolic reprograming is a key feature of the innate immune memory phenotype. Under steady-state conditions, immune cells exhibit low biosynthetic activity and rely on OXPHOS and FAO to meet energy requirements. Upon activation innate immune cells experience heightened energy demands, which are met by upregulating glycolysis, the TCA cycle and glutaminolysis, which facilitate antimicrobial functions including phagocytosis and microbial killing.^14,37–39^ In addition to fueling immune responses, the accumulation of metabolic intermediates such as acetyl-CoA, fumarate, succinate, nicotinamide adenine dinucleotide (NAD+) and mevalonate, plays a critical role in reshaping the epigenetic landscape of immune cells. These intermediates act as substrates or cofactors for epigenetic enzymes, influencing chromatin remodeling and transcriptional reprograming, both of which are essential for establishing the trained immune phenotype^40^ (Fig. 2).

### 1.3 Glycolysis

Glycolysis is significantly upregulated during immune cell activation to fulfill the rapid energy and biosynthetic demands essential for robust immune responses.^41,42^ Beyond generating energy, glycolysis serves as a critical source of intermediates for biosynthetic pathways, enabling the metabolic reprograming required for immune cell activation and response to external stimuli.^14^ A metabolic shift toward glycolysis with reduced reliance on OXPHOS was first observed in β-glucan-trained human monocytes. It plays a pivotal role in enhancing immune resistance to systemic infections.^43^ Inhibition of glycolysis with a PFKFB3 inhibitor or 2-deoxyglucose (2-DG) dampens the heightened cytokine responsiveness in trained leukocytes without affecting untrained cells.^10,44^ The glycolytic reprograming associated with innate immune memory is driven by the activation of the Akt/mTOR/Hif1*α* signaling pathway, a pattern also observed in monocytes exposed to BCG or oxidized low-density lipoprotein (oxLDL).^45^ Blocking hypoxia-inducible factor 1alpha (HIF-1*α*) with ascorbate diminishes pro-inflammatory cytokine production in β-glucan- or BCG-treated monocytes, demonstrating the reliance of trained cells on this pathway.^43^ Similarly, myeloid-specific deletion of *HIF1A* compromises the survival benefit conferred by β-glucan training during *S. aureus* infection. Sustained HIF-1*α*-dependent glycolysis also drives enhanced phagocytosis in MPLA-trained macrophages.^11^ Furthermore, inhibition of the mammalian target of rapamycin (mTOR) disrupts glycolytic reprograming induced by MPLA- and CpG-ODN, reduces mitochondrial respiration, and prevents increased phagocytic capacity and cytokine production.^10,46^ Rapamycin administration completely abolishes the survival benefit conferred by MPLA treatment during systemic *S. aureus* infection in mice.^10^ Additionally, neutrophils from BCG-vaccinated mice exhibit increased glycolysis upon *C. albicans* stimulation, correlating with enhanced microbial killing.^47^

The bilateral relationship between the upregulation of glycolytic metabolism and epigenetic reprograming is pivotal in innate immune memory. Glycolysis supports the persistence of the trained phenotype by activating histone modifications, such as histone H3 trimethyl lysine 4 (H3K4me3), at cytokine and metabolic gene promoters.^44^ For example, oxLDL-trained macrophages exhibit increased H3K4me3 at *PFKFB3* and *HK2* promoters, an effect diminished when glycolysis is inhibited by 2-DG.^48^ Changes in the NAD+/NADP ratio, which is elevated in trained macrophages due to enhanced glucose consumption and lactate production, are common feature of trained innate leukocytes. This metabolic shift activates NAD + -dependent enzymes like sirtuins, which remove lysine acetyl-groups from histones.^49^ Inhibition of NAD-dependent histone deacetylases with resveratrol reduces β-glucan-induced cytokine production.^43^ Conversely, histone modifications also regulate glycolytic enzyme expression, as methyltransferase inhibitors like methylthioadenosine or the Set7 inhibitor cyproheptadine prevent sustained glycolytic upregulation.^43,44^ These findings highlight the reciprocal regulation between glycolytic metabolism and epigenetic reprograming as mechanisms driving innate immune memory.

In summary, evidence supports that enhancement of glycolysis, driven by activation of the Akt/mTOR/Hif1*α* pathway, is essential for metabolic reprograming and the augmented antimicrobial phenotype associated with innate immune memory. The metabolic shift toward glycolysis not only provides the necessary energy and biosynthetic precursors for immune activation, but also supports long-term immune memory through epigenetic modifications. Better understanding of the dynamic interplay between these metabolic and epigenetic pathways not only deepens knowledge but has the potential to uncover therapeutic targets for modulating innate immune responses.

### 1.4 Oxidative phosphorylation

OXPHOS is a mitochondrial process relying on proton gradients to generate ATP efficiently. The TCA-generated metabolites NADH and flavin adenine dinucleotide (FADH2) supply electrons to the electron transport chain (ETC), which then drives proton pumping across the inner mitochondrial membrane. This proton gradient powers ATP synthase to produce ATP.^50^ As discussed above, stimuli like β-glucan induce a metabolic shift toward increased glycolysis and reduced OXPHOS, a signature of the Warburg effect.^43^ Monocytes trained with BCG or oxLDL induce a similar metabolic shift.^45^ Like monocytes, trained macrophages show sustained glycolysis. Yet, trained macrophages also exhibit augmented oxidative metabolism.^10^ Increased TCA cycle flux, mitochondrial mass and mitochondrial membrane potential parallel increased oxidative metabolism.^51^ Importantly, disrupting mitochondrial function or oxidative metabolism pharmacologically reduces the memory phenotype induced by oxLDL, MPLA, or β-glucan.^10,52^ Single nucleotide polymorphisms in genes encoding ETC complexes I, II, and IV correlate with the ability of oxLDL-trained PMBCs to produce TNFα and IL-6 following LPS restimulation,^48,51^ highlighting the contribution of reprogramed OXPHOS in sustaining the memory phenotype.

Metformin, a mitochondrial complex I inhibitor, blocks the development of the memory macrophage phenotype when co-administered with β-glucan or oxLDL. This effect may also result from metformin's inhibition of mTOR signaling through activation of adenosine triphosphate (ATP) dependent protein kinase.^43^ Moreover, oligomycin, an ATP synthase inhibitor, prevents increased cytokine production in MPLA-trained macrophages^10^ and in β-glucan-trained monocytes during LPS restimulation,^48^ suggesting that OXPHOS-derived energy production is required for the memory phenotype. However, in the context of BCG-induced training, oligomycin does not inhibit the induction of augmented cytokine production,^53^ suggesting that OXPHOS requirements may vary depending on the training stimulus.

Increased OXPHOS during trained immunity not only supportsATP production but also generates reactive oxygen species (ROS), which are crucial for killing of pathogens. Studies indicate that oxLDL training significantly elevates cytosolic and mitochondrial ROS formation in human monocytes through an mTOR-dependent mechanism.^45,51,54^ ROS formation is pivotal for establishing the memory phenotype, including increased cytokine production, mTOR phosphorylation, HIF-1*α* stabilization, and lactate production.^54^ Elevated ROS production has also been observed in BCG-trained human monocytes,^51^ while β-glucan-trained monocytes demonstrate reduced ROS production.^51^ In contrast, macrophages trained with β-glucan, CpG or MPLA exhibit increased ROS production.^9,10,46^ These findings highlight the complexity of ROS regulation during innate immune memory and underscore the need for further research to elucidate the precise role of ROS. Collectively, the coordinated metabolic shift toward glycolysis and OXPHOS is an important driver of the memory phenotype. Understanding the interplay between these metabolic processes and their regulation by different training stimuli could yield novel strategies for modulating innate immune responses.

### 1.5 Pentose phosphate pathway

**Fig. 3.**
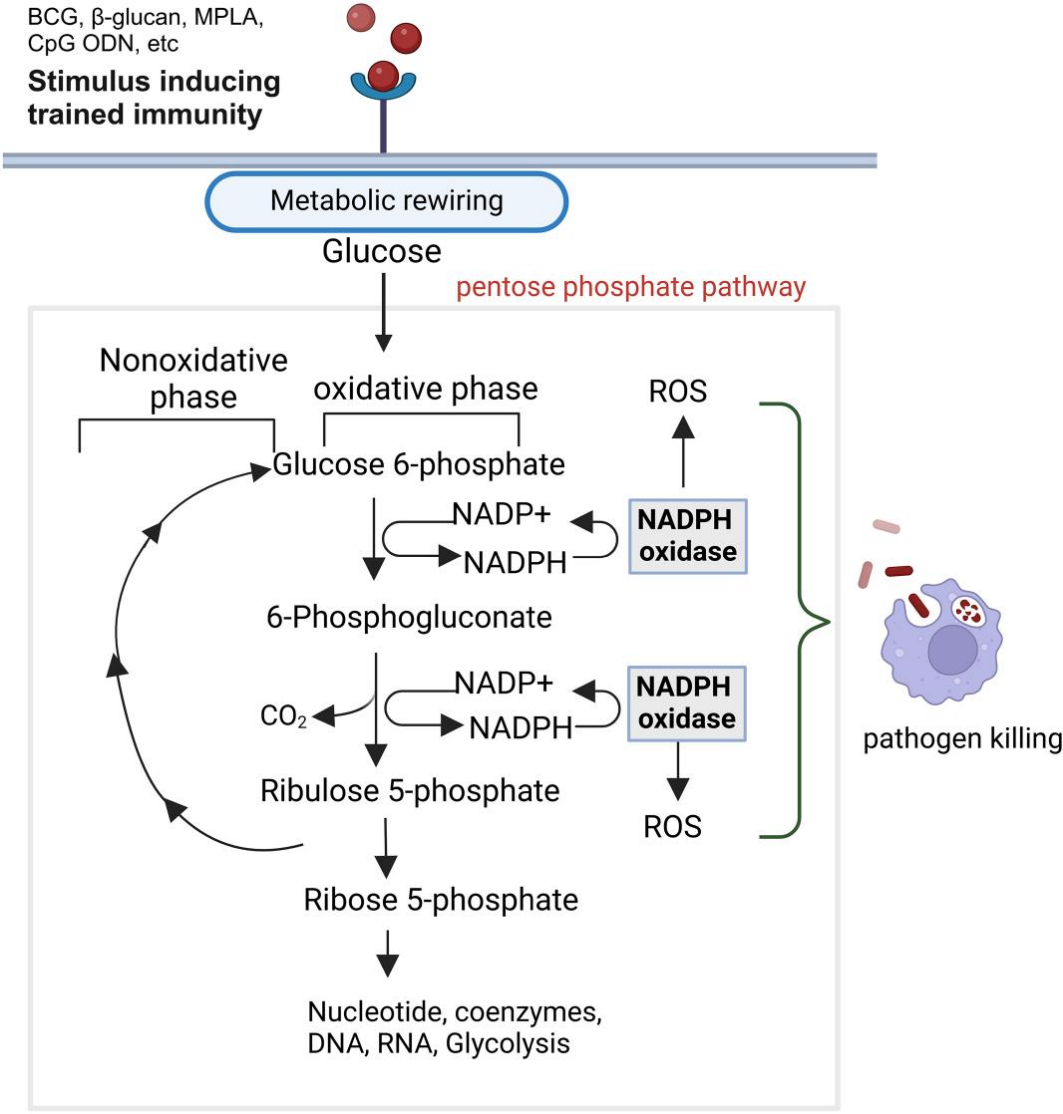
Potential contribution of the PPP to innate immune memory. The PPP is a glucose-oxidizing pathway that runs in parallel to glycolysis, generating NADPH through its oxidative branch. It may be essential for the persistence of trained immunity by facilitating pathogen killing through NADPH oxidase-driven ROS production. NADPH, nicotinamide adenine dinucleotide phosphate; ROS, reactive oxygen species.

The pentose phosphate pathway (PPP) is important for the production of amino acid precursors and nucleotides, both essential for cellular growth and proliferation (Fig. 3). Beyond its biosynthetic contributions, the PPP generates NAD+ phosphate (NADPH), a key molecule for maintaining redox homeostasis and biosynthetic processes, such as the synthesis of tetrahydrofolate, deoxyribonucleotides, proline, fatty acids and cholesterol. Importantly, PPP-derived NADPH supports the generation of ROS and reactive nitrogen species, which are essential for cellular signaling and killing of microbes.^50^

Activation of the oxidative PPP (OxPPP), is essential for generating NADPH to support the oxidative burst and other effector functions of neutrophils, ultimately allowing them to kill pathogens.^55^ Consistently, deficiencies in either oxPPP enzyme G6PD or the non-OxPPP enzyme TALDO, are associated with increased susceptibility to recurrent infections and sepsis in affected patients.^56,57^ In BCG-induced training of human monocytes, the increased incorporation of ^13^C-labels was increased in ribosyl-1, suggesting activation of the oxidative branch of the PPP. However, pharmacological blockade of this branch does not interfere with BCG-induced TNF and IL-6 production, suggesting that PPP activation may not be critical for cytokine production during training.^53^ Nevertheless, it is reasonable to hypothesize that the PPP contributes to the persistence of innate immune memory in vivo by facilitating killing of pathogens via NADPH oxidase-driven ROS production. Additionally, the PPP may play a role in supporting the proliferation of myeloid progenitor cells, thus sustaining trained immune responses. However, these potential functions remain unexplored and warrant further investigation.

### 1.6 TCA cycle

The TCA cycle is a core mitochondrial process that facilitates a series of oxidative reactions, reducing NADH and FADH2 coenzymes, which subsequently fuel the ETC and ATP production.^58^ Beyond its role in energy production, the TCA cycle integrates multiple anabolic and catabolic pathways, generating key metabolic intermediates that support cellular functions.^38^ Accumulation of TCA cycle intermediates, replenished through two primary carbon sources, supports innate immune memory. Pyruvate-derived acetyl-CoA, a product of glycolysis that reacts with oxaloacetate to initiate the TCA cycle via citrate biosynthesis, and acetyl-CoA derived from the *β*-oxidation of fatty acids are major drivers of the TCA cycle.^59^ The second key carbon source is glutamine, which enters the TCA cycle after conversion into *α*-ketoglutarate (*α*-KG) through glutaminolysis. Arts et al. found that glutamine-derived *α*-KG fuels increased intracellular fumarate levels, a key metabolite for inducing trained immunity.^60^ Inhibition of glutaminolysis disrupts the heightened responsiveness of β-glucan-trained macrophages, highlighting the necessity of replenishing the TCA cycle via glutaminolysis to sustain trained immunity.

In addition to its metabolic role, the TCA cycle supports processes that influence cellular function and immune responsiveness. By generating both energy and essential intermediates, the TCA cycle facilitates the metabolic and epigenetic reprograming that underlies trained immunity, highlighting the indispensable role of mitochondrial metabolism in shaping innate immune memory.

### 1.7 TCA cycle metabolites

The induction of innate immune memory hinges on the accumulation and functional modulation of TCA cycle intermediate metabolites. Fensterheim et al. showed a break in the TCA cycle distal to citrate in trained bone marrow-derived macrophages, with reestablished flux at α-ketoglutarate (αKG) via glutamine anapleurosis.^10^ The break distal to citrate is associated with increased production of itaconate and citrate export from mitochondria into the cytoplasm, leading to extramitochondrial acetyl-CoA accumulation. Itaconate, derived from *cis*-aconitate via the enzyme immune-responsive gene 1 (Irg1), has several functions.^61–63^ It inhibits succinate dehydrogenase (SDH) by occupying the binding pocket of succinate,^35^ leading to succinate accumulation, which limits excessive ROS and pro-inflammatory cytokine production.^61^ Additionally, itaconate directly inhibits isocitrate lyase, impairing the bacterial glyoxylate shunt^64^ critical for bacterial survival.^65–67^ Notably, a recent study showed that MPLA treatment facilitates itaconate accumulation at sites of infection, augmenting microbial clearance—a response diminished in Irg1-deficient mice.^68^ Further studies show that itaconate accumulates in trained macrophages and is directly antimicrobial against *P. aeruginosa* within the acidic phagolysosome environment, an effect ie facilitated by ROS.^68^ Conversely, Dominguez-Andres et. al. showed that β-glucan inhibits Irg1 expression in a model of human endotoxemia, reversing endotoxin tolerance, increasing SDH expression, and enhancing the innate immune response to secondary stimulus.^69^ Dimethyl itaconate also induces metabolic reprograming in innate leukocytes, providing protection against infection.^70^ Collectively, these studies indicate a role of itaconate in regulating the innate memory phenotype.

Succinate, malate and fumarate also accumulate in trained innate leukocytes.^10,60^ Elevated levels of succinate and fumarate in trained monocytes act as competitive inhibitors of multiple *α*-KG dependent dioxygenases (*α*-KGDD), including histone and DNA demethylases such as the Jumonji (Jmj)-KDM5 family. This inhibition prevents the removal of histone trimethylation marks, such as H3K4me3 and H3K27me3,^71^ which are crucial for maintaining pro-inflammatory gene expression. Monomethyl fumarate increases the responsiveness of human monocytes to inflammatory stimuli by enriching H3K4me3 marks at the promoter regions of pro-inflammatory genes, such as *TNFA* and *IL6*, while also inhibiting the activity of the demethylase KDM5.^60^ Additionally, succinate and fumarate stabilize HIF1α by inhibiting prolyl hydroxylases, promoting IL-1β transcription and glycolysis.^72^ HIF1α induces the expression of histone demethylase expression,^73^ linking TCA cycle intermediates to epigenomic remodeling.

Mitochondrial SDH, also known as complex II, catalyzes the conversion of succinate to fumarate while transferring electrons to the ETC. β-glucan-trained monocytes show increased expression of various SDH subunits.^69^ Human genomic studies have identified SNPs in SDH-coding genes that correlate with variations in inflammatory responsiveness.^48,69^ The lysine methyltransferase Set7 regulates SDH gene transcription, with pharmacological or genetic inhibition of Set7 decreasing the upregulation of SDHB transcript in β-glucan-trained leukocytes.^48^ Malate metabolism adapts dynamically in trained macrophages. For example, MPLA-trained macrophages initially utilize aerobic glycolysis, converting malate to pyruvate via cytosolic malic enzyme 1 for ATP production. Three days after MPLA exposure, these cells restore malate shuttling to channel NADH into the mitochondria, supporting ATP synthesis through OXPHOS.^10^ These metabolic adaptations underscore the centrality of TCA cycle intermediates in the metabolic and epigenetic reprograming of innate immune memory.

### 1.8 Lipid metabolism

Transcriptome and metabolome analyses of β-glucan-trained macrophages reveal upregulation of cholesterol and fatty acid synthesis pathways.^53,74–76^ Cholesterol synthesis plays a vital role in trained immunity, as inhibiting HMG-CoA reductase, the rate-limiting enzyme in this pathway, impairs trained immunity induced by β-glucan, oxLDL, or BCG.^53,74^ This inhibition disrupts lactate production and glycolysis, as well as cytokine production and epigenetic reprogramming, as evidenced by reduced enrichment of H3K4me3 at cytokine gene promoters.^74^ The accumulation of mevalonate, a key intermediate in cholesterol synthesis, amplifies trained immunity through the IGF1R-Akt-mTOR pathway, promoting histone modifications associated with inflammatory pathways.^74^ Monocytes from hyper-IgD syndrome patients, who have a mevalonate kinase deficiency, exhibit a constitutive trained immunity phenotype at both immunological and epigenetic levels. Moreover, statin-mediated inhibition of the cholesterol synthesis pathway prevents the innate immune memory phenotype.^74^

Fatty acid synthesis represents a key metabolic adaptation in innate immune memory. While typically associated with a pro-inflammatory macrophage phenotype, fatty acids also play a role in regulating intracellular stress and innate immune pathways. β-Glucan-trained macrophages exhibit upregulated fatty acid synthesis; however, pharmacological inhibition of this pathway during β-glucan exposure does not interfere with trained immunity.^60^ In contrast, fatty acid synthesis is essential in aldosterone-induced trained immunity.^77^ In aldosterone-trained macrophages, restimulation with the TLR 2 ligand Pam3Cys enhances fatty acid synthesis, accompanied by increased H3K4me3 enrichment at promoter regions of key genes. Inhibition of fatty acid synthesis in this context abolishes the enhanced cytokine production,^77^ emphasizing its role innate immune memory.

Recent studies demonstrate that human monocytes exposed to BCG accumulate lipid mediators derived from long-chain polyunsaturated fatty acids. Enzymes such as fatty acid desaturase and lipoxygenase (LOX) are critical for the establishment of BCG-induced trained immunity, as their inhibition decreases BCG-induced pro-inflammatory cytokine production.^78^ Lipid metabolites, including products of 12-LOX activity, are elevated in monocytes of healthy individuals following BCG vaccination.^78^ These findings highlight the central roles of cholesterol and fatty acid metabolism in shaping and maintaining trained immunity.

### 1.9 Targeting trained immunity for therapeutic benefits

Trained immunity modulates immune responses across various disease states, influencing both protective immunity and pathological inflammation.

### 1.10 Boosting vaccine effectiveness and nonspecific immunity

**Fig. 4.**
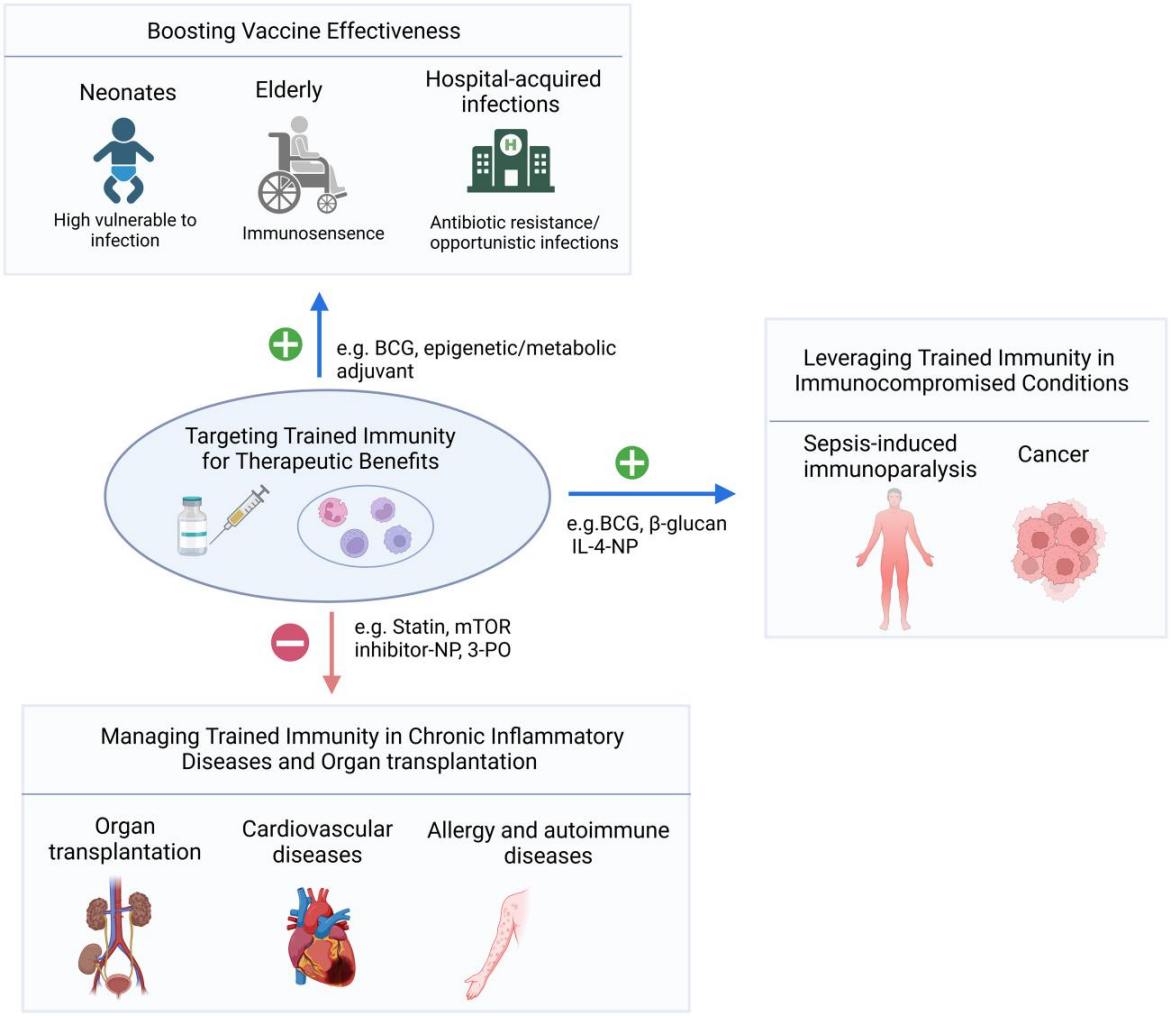
Targeting trained immunity for therapeutic benefits. Modulation of innate immune memory holds therapeutic potential across various clinical contexts. Enhancing innate immunity can improve vaccine efficacy, offering both pathogen-specific protection and cross-protection against unrelated infections. This strategy is particularly beneficial in regions with high infection burdens, in the fight against antibiotic-resistant pathogens, and for boosting immunity in vulnerable populations, such as neonates and the elderly. Additionally, strengthening innate immune responses may benefit immunocompromised individuals, such as those with sepsis or cancer. Conversely, reducing innate immune memory may provide therapeutic benefits in conditions with excessive immune activation, including cardiovascular diseases and graft-vs-host disease. BCG, Bacillus Calmette–Guerin; NP, nanoparticles; IL-4, interleukin-4; 3PO, 3-[3-pyridinyl]-1-[4-pyridinyl]-2-propen-1-one; mTOR, the mammalian target of rapamycin.

Many immune training reagents, including MPLA and CpG-ODN, serve as adjuvants in commercially produced and widely distributed vaccines. MPLA is a component of vaccines targeting shingles (Shingrix), human papilloma virus (Cervarix) and hepatitis B (Fendrix).^79^ Vaccines targeting hepatitis B (Heplisav-B) and COVID-19 contain CpG-ODN.^80^ Both reagents strongly augment the efficacy of vaccines, especially in elderly populations (Fig. 4).

The BCG vaccine, initially developed for tuberculosis, serves as the prototypical example of a vaccine that induces trained immunity to reduce mortality from nontuberculosis infection.^81,82^ Clinical trials in adults^83^ and children^84,85^ demonstrated that BCG induces nonspecific activation of innate immune cells. Additionally, BCG vaccination provides protection against microorganisms in controlled human infection models, such as yellow fever^86^ and malaria,^87^ through epigenetic and metabolic reprograming of innate immune cells. Recent studies indicate that BCG can also induce trained immunity in the elderly.^88,89^ Yet, we need further exploration to determine whether and how aging impacts trained immunity in the context of endogenous stimuli. This evidence highlights trained immunity as a promising strategy to enhance vaccine efficacy in vulnerable populations, including neonates and the elderly. It also presents an avenue to boost innate immunity in individuals at high risk for hospital-acquired infections, potentially mitigating antibiotic resistance.^90^

### 1.11 Leveraging trained immunity in immunocompromised conditions

Trained immunity may benefit immunocompromised patients, such as those with sepsis or cancer (Fig. 4). Sepsis-induced immunoparalysis, characterized by a persistent anti-inflammatory response, increases susceptibility to opportunistic infections and mortality.^91^ A recent preclinical study demonstrated that nanoparticle-mediated IL-4 delivery to myeloid cells restores immune balance via trained immunity in an ex vivo human sepsis model.^92^ This approach holds translational potential, offering a novel therapeutic strategy for rebalancing immune responses in sepsis. Further research is required to validate these findings and explore clinical applications.

In addition, BCG vaccination has shown antitumor effects in malignancies such as bladder cancer,^93^ melanoma,^94^ leukemia^95^ and lymphoma,^96^ primarily through trained immunity in monocytes and macrophages.^97^ Moreover, *β*-glucan trafficking to the pancreas promotes a CCR2-dependent monocyte and macrophage influx, stimulating antitumor activity to slow pancreatic cancer growth.^98^ However, excessive activation of innate immunity within the tumor microenvironment can foster chronic inflammation and immune evasion, emphasizing the need for precise regulation of trained immunity to optimize therapeutic outcomes.

### 1.12 Managing trained immunity in chronic inflammatory diseases

Conversely, maladaptive trained immunity contributes to chronic inflammatory conditions such as cardiovascular, autoimmune and autoinflammatory diseases, as well as complications in organ transplantation (Fig. 4). In these contexts, dampening innate immune memory may offer a potential therapeutic strategy. Inhibition of certain metabolic pathways involved in trained immunity presents an attractive approach. Rapamycin-loaded HDL nanoparticles effectively inhibit Akt-mTOR pathway, suppressing trained immunity induced by allograft transplantation and preventing allograft rejection.^99^ In atherosclerosis, intravenous infusion of statin^100,101^ or mTOR inhibitor^102^ based nanobiologics dramatically reduced vessel wall inflammation in ApoE-/− mice. Additionally, partial inhibition of glycolytic flux using the small molecule 3PO (3-[3-pyridinyl]-1-[4-pyridinyl]-2-propen-1-one) has been shown to mitigate atherosclerosis progression in a mouse model, as demonstrated by Perotta et al.^103^

Further research is essential to elucidate the interplay between trained immunity, disease pathologies, and intracellular metabolic and epigenetic reprograming. Investigating drugs that modulate immune responses, metabolism, and epigenetic enzymes will be critical in harnessing trained immunity for therapeutic benefit.

## 2. Conclusion

Innate immune memory results from long-lasting reprograming of innate immune cells, enhancing their capacity to respond to subsequent challenges. This phenomenon holds significant therapeutic potential in immunosuppressed patients, such as those with cancer, critical illness, diabetes, premature birth (neonates), multiple co-morbidities and advanced age. It is particularly relevant in mitigating immunoparalysis associated with sepsis or trauma, which often involves profound metabolic impairments in innate leukocytes. Addressing these metabolic disturbances may help restore immune functionality and improve clinical outcomes.^104,105^

Complex functional, epigenetic, and metabolic alterations govern the establishment and persistence of innate immune memory. Among these, metabolic remodeling emerges as a central driver. Alterations in metabolic pathways not only supply the energy and substrates necessary for cellular functions but also drive epigenetic modifications by regulating chromatin-modifying enzymes. This review highlights the role of metabolic adaptations in shaping innate immune memory, encompassing key pathways such as glycolysis, OXPHOS, glutaminolysis, the TCA cycle, and cholesterol and fatty acid synthesis. These metabolic processes provide essential metabolites and energy to support the functional and epigenetic adaptations central to innate immune memory. Despite significant progress in this field, several critical questions remain unanswered. Key areas for further exploration include elucidating the roles of PPP and ROS in driving the antimicrobial phenotype induced by innate immune training reagents.

Therapeutic targeting of these metabolic pathways presents unique challenges, requiring precise modulation to avoid adverse outcomes. Sustained activation of innate immune memory mechanisms carries the risk of chronic inflammatory conditions, such as arthritis, lupus or atherosclerosis. Therefore, tailored strategies are essential for targeting these metabolic pathways in a time- and site-specific manner to minimize unintended side effects and maximize therapeutic efficacy. Continued investigation is essential for developing precise, metabolism-based therapeutic strategies for diverse clinical challenges; fully leveraging the therapeutic potential of innate immune memory while mitigating associated risks.
